# High-Level Visual Encoding Model Framework with Hierarchical Ventral Stream-Optimized Neural Networks

**DOI:** 10.3390/brainsci12081101

**Published:** 2022-08-19

**Authors:** Wulue Xiao, Jingwei Li, Chi Zhang, Linyuan Wang, Panpan Chen, Ziya Yu, Li Tong, Bin Yan

**Affiliations:** 1 School of Cyber Science and Engineering, Zhengzhou University, Zhengzhou 450001, China; 2 Henan Key Laboratory of Imaging and Intelligent Processing, PLA Strategic Support Force Information Engineering University, Zhengzhou 450001, China

**Keywords:** fMRI, encoding model, deep neural networks, ventral stream, hierarchical representations

## Abstract

Visual encoding models based on deep neural networks (DNN) show good performance in predicting brain activity in low-level visual areas. However, due to the amount of neural data limitation, DNN-based visual encoding models are difficult to fit for high-level visual areas, resulting in insufficient encoding performance. The ventral stream suggests that higher visual areas receive information from lower visual areas, which is not fully reflected in the current encoding models. In the present study, we propose a novel visual encoding model framework which uses the hierarchy of representations in the ventral stream to improve the model’s performance in high-level visual areas. Under the framework, we propose two categories of hierarchical encoding models from the voxel and the feature perspectives to realize the hierarchical representations. From the voxel perspective, we first constructed an encoding model for the low-level visual area (V1 or V2) and extracted the voxel space predicted by the model. Then we use the extracted voxel space of the low-level visual area to predict the voxel space of the high-level visual area (V4 or LO) via constructing a voxel-to-voxel model. From the feature perspective, the feature space of the first model is extracted to predict the voxel space of the high-level visual area. The experimental results show that two categories of hierarchical encoding models effectively improve the encoding performance in V4 and LO. In addition, the proportion of the best-encoded voxels for different models in V4 and LO show that our proposed models have obvious advantages in prediction accuracy. We find that the hierarchy of representations in the ventral stream has a positive effect on improving the performance of the existing model in high-level visual areas.

## 1. Introduction

An important aspect of cognitive neuroscience is modeling the human brain’s response to external stimuli and predicting the corresponding brain activity. Models that predict the brain’s response to external stimuli are known as visual encoding models [1,2]. Currently, some DNN-based visual encoding models have achieved the best predictive performance. DNN was initially inspired by how the brain’s feedforward visual system processes visual information [3]. Here, DNN-based visual encoding models assume that the voxels activity in the brain can be modeled as a linear combination of the activity artificial neurons in DNN. According to different driving modes, existing DNN-based visual encoding models can be divided into two categories: goal-driven visual encoding models and data-driven visual encoding models.

Goal-driven visual encoding models can accurately predict the voxel activity in the ventral and dorsal visual areas [4,5] based on the visual stimuli features extracted from pre-trained DNN [6,7,8,9,10,11,12]. Moreover, there is a hierarchical correspondence between the DNN and the human visual system: the features from the lower level net layers can better predict the activity of the early visual cortex, while the higher level net layers can better predict the later visual cortex [13,14,15]. However, the features learned by DNN are not directly optimized by fitting functional magnetic resonance imaging (fMRI) data but are ultimately optimized to serve a certain high-level vision task (e.g., image recognition) in computer vision [16,17,18]. This is different from the perceptual mechanism of the human visual system, which performs multiple visual tasks. In addition, there are still many differences between DNN and the human visual system. For example, the learning process of most DNN is supervised. In contrast, the human visual system is unsupervised [19,20]; DNN relies on local texture and shape features [21,22], while the human visual system considers both local and global shape contour features [23,24,25]. Therefore, the features from pre-trained DNN are hard matched to fMRI data, resulting in the features not being suitable for encoding brain activity in high-level visual areas.

Data-driven visual encoding models can realize the nonlinear mapping from the visual stimuli to the brain response and learn the representation directly from the fMRI data in an end-to-end manner [26,27,28,29,30,31,32]. For example, Seeliger et al. [31] proposed an end-to-end encoding model that simultaneously represents the neural information processing between different visual cortex and shows good fitting ability on the voxel activity of early visual areas. Existing works show that the end-to-end encoding model has obvious advantages in low-level visual areas and can learn effective representation directly from the neural data [30,32]. However, the amount of fMRI data is usually small, making it difficult for models to learn effective representations for complex and abstract high-level visual areas, resulting in insufficient encoding performance.

Many works in neuroscience have shown that the primate brain processes visual information hierarchically [33,34,35]. In the ventral stream, after the visual information reaches the primary visual cortex V1, it is passed upward through the secondary visual cortex V2 to the high-level visual cortex V4 and beyond (e.g., lateral occipital, LO), where the representation of the information becomes more complex. The hierarchy of representations in the ventral stream suggests that the higher visual cortex receives information from the lower visual cortex. In addition, existing research explored the mechanism of visual information processing in the brain by constructing relevant computational models [36,37,38,39,40,41,42], including for a single visual area [37,41] and information flow across visual areas [36,38,39,40]. Recently, Mell et al. [43] realized the mutual prediction between voxels in different visual areas using constructed voxel-to-voxel model achieving higher prediction accuracy than the DNN-based visual encoding model. These existing works show that there is interaction and information exchange between visual areas. However, the hierarchical representation in the ventral stream and the interaction between visual areas is not fully reflected in the existing encoding models.

In this paper, we proposed a high-level visual encoding model framework with hierarchical ventral stream-optimized neural networks, which is different from existing encoding models in which the representation space of the model is optimized globally based on brain activity in a single visual area. The encoding strategy of our framework fully reflects the interaction between visual areas from the perspective of the hierarchy of representations, and the representation space of the model is optimized based on low-level and high-level visual areas successively. Under the framework, we introduced two categories of bottom-up hierarchical information convey methods and built corresponding encoding models. The representation space is initially obtained based on different low-level visual areas, then selectively conveyed to a specific high-level visual area for further optimization to improve the model’s performance. The first method conveys hierarchical information from the voxel perspective; we constructed an encoding model from stimulus to voxels of the low-level visual area and then to voxels of the high-level visual area (S2V2V-EM). The second method conveys hierarchical information from the feature perspective; we constructed an encoding model from stimulus to low-level image features and then to voxels of the high-level visual area (S2F2V-EM). In comparison with control models, both S2V2V-EM and S2F2V-EM achieved better encoding performance in the V4 and LO. In addition, we calculated the proportion of best-encoded voxels for different encoding models in V4 and LO, and experimental results show that S2V2V-EM and S2F2V-EM have obvious advantages in the prediction accuracy of voxel activity. These results show that using the hierarchy of representations in the ventral stream to improve the performance of the currently available encoding model is an effective strategy. In this manuscript, we combined neuroscience with deep learning and expect to promote the understanding of the brain’s visual system and the development of brain-inspired intelligence.

## 2. Materials and Methods

### 2.1. fMRI Data

In this paper, fMRI data were used from the publicly available vim-1 dataset (data are available at https://crcns.org/data-sets/vc/vim-1 (accessed on 27 June 2022)). Details of the experimental design, MRI acquisition protocol, and preprocessing of the data in this dataset can be found in previous studies [44,45]. Briefly, the dataset contains the blood oxygenation level–dependent (BOLD) activity of the brain’s visual cortex in two healthy male subjects (S1 and S2) viewing 1870 grayscale natural scene images (20 × 20°). Images were presented in successive 4 s trials. In each trial, a picture was flashed for 1s (with 200 ms as interval, with the sequence ON-OFF-ON-OFF-ON), followed by a gray background picture for 3s. fMRI data were collected using a 4T INOVA MR scanner (Varian, Inc., Palo Alto, CA, USA). Data were collected from 18 coronal slices covering covered occipital cortex (slice thickness 2.25 mm, slice gap 0.25 mm, field of view 128 mm × 128 mm). Functional data were acquired using a gradient-echo EPI (echo-planar imaging) pulse sequence (TR 1000 ms, TE 28 ms, matrix size 64 × 64, flip angle 20°, spatial resolution 2 mm × 2 mm × 2.5 mm).

Each stimulus image was matched with the voxel activity evoked in the subject viewing the image and formed a sample pair. For each subject, the data contains 1750 pairs samples of the training set and 120 pairs samples of the validation set. We selected the voxel activity in V1, V2, V4 and LO areas of S1 in this dataset for further analysis.

### 2.2. Encoding Model Framework

#### 2.2.1. General Encoding Model from Stimulus to Voxel

**Figure 1:**
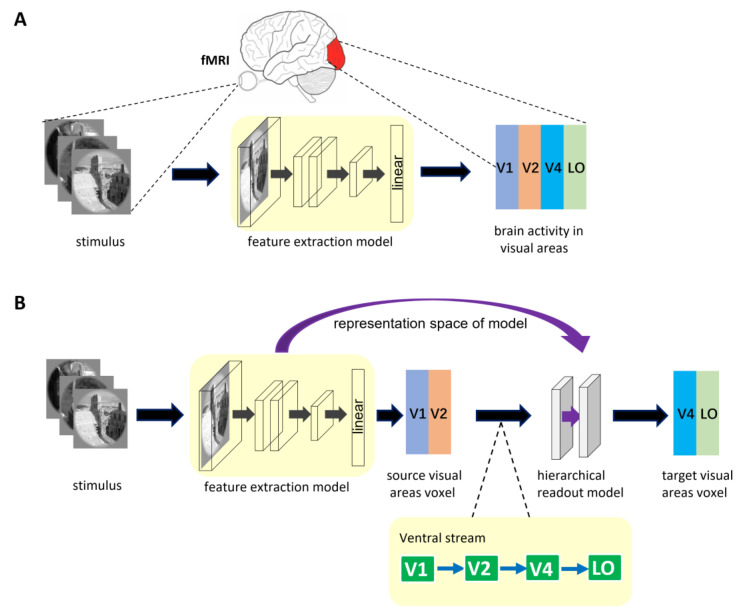
The described encoding model architecture. (**A**) The general encoding model from stimulus to voxel. In the model, the feature extraction model is trained separately for different visual areas. (**B**) Encoding model with hierarchical ventral stream-optimized neural networks. The representation space of the S2V-EM trained on the source visual area is extracted. Then, the representation space is optimized again in the hierarchical readout model to predict the voxel activity of the target visual area. The bottom of the figure is shown as the hierarchy of representations in the ventral visual stream. The ventral visual stream is represented by the connection of the blue direction arrow.

The architecture of general encoding models from stimulus to voxel (S2V-EM) is shown in Figure 1A. The upper part of the figure shows the brain activity of visual areas obtained via fMRI in the human subject viewing visual stimulus. The process of visual encoding can be roughly divided into two stages. The stimulus images are first fed into the feature extraction model (e.g., a pre-trained DNN model), and the feature space of the stimulus images is obtained after a series of nonlinear calculations in the model. Then, the feature space is used to predict the voxel space of the corresponding visual area via linear readout layer. In general, the representation space of S2V-EM is optimized based on single computer vision tasks or voxel activity in a single visual area. The representation space is used to share encoding between different visual areas or independent encoding a specific visual area. However, a disadvantage is that these models’ performance is bad in high-level visual areas.

#### 2.2.2. Encoding Model with Hierarchical Ventral Stream-Optimized Neural Networks

The hierarchy of representations in the ventral stream shows that the higher visual cortex contains information from the lower visual cortex. Based on the mechanism, we propose a high-level visual encoding model framework with hierarchical ventral stream-optimized neural networks. The framework architecture is shown in Figure 1B. Different from general S2V-EM, the encoding model based on this framework is not constructed by directly fitting the high-level visual area but is constructed based on the bottom-up hierarchical representation strategy. Specifically, we first built an encoding model for the low-level visual area, then extracted the fitting information of the model as the intermediate state of hierarchical representation, and finally completed the encoding from the low-level visual area to the high-level visual area. Encoding models under the framework consist of two sub-models and use the hierarchy of representations in the ventral visual stream as a constraint. The first sub-model is a general S2V-EM. Under the framework, we mainly used the S2V-EM to realize the encoding for low-level visual areas (i.e., source visual areas). The second sub-model is the hierarchical readout model, which implements the process of bottom-up hierarchical information conveyance. We extracted the representation space of the S2V-EM as the input of the hierarchical readout model and finally used it to predict the voxel space of the target visual area. In addition, for the specific source visual area and target visual area, we used the known topological connections of the ventral visual areas [46] as constraints. Take the target visual area V4 as an example; we extracted the representation space of the S2V-EM that is trained in the source visual area V1 or V2, and then applied the hierarchical readout model to realize the V1→V4 and V2→V4 encoding, respectively.

In this paper, based on the framework, we constructed two categories of hierarchical encoding models (S2V2V-EM and S2F2V-EM) from the voxel and the feature perspectives to achieve hierarchical representation in the ventral stream. The detailed introduction to S2V2V-EM and S2F2V-EM can be found in Section 2.3. 

### 2.3. Predictive Models

#### 2.3.1. S2V-EM

For the S2V-EM in the framework, we used an encoding model based on the fusion features of Gabor and deep neural network (GaborNet-VE). The model can be trained end-to-end on the vim-1 dataset from stimulus images to voxel activity. Full details regarding this model can be found in Cui et al. [32].

**Figure 2:**
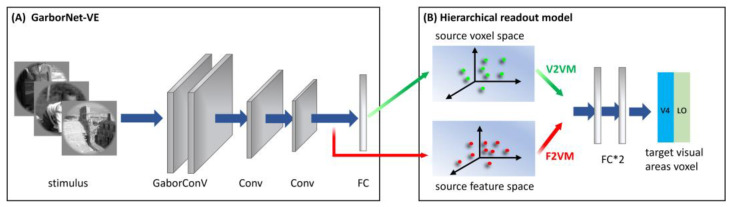
The architecture of S2V2V-EM and S2F2V-EM. (**A**) Simplified structure of GaborNet-VE. The model consisted mainly of one Gabor convolutional layer, two regular convolutional layers and one fully connected layer (FC). (**B**) Hierarchical readout model. Including the voxel-to-voxel model and the feature-to-voxel model. In the model, the voxel space (or feature space) of the GaborNet-VE is extracted to predict the voxel space of the high-level visual area V4 or LO. Green and red direction arrows indicate hierarchical information conveyed from the voxel and the feature perspectives, respectively.

Briefly, the GaborNet-VE consists mainly of a Gabor convolutional layer, two regular convolutional layers, and one fully connected layer (see Figure 2A). The Gabor convolutional layer contains 128 Gabor kernels, including 64 Gabor kernels of the real and imaginary types. The size of each Gabor kernel is 9 × 9. The model realizes the learning of deep network features based on Gabor features via replacing regular convolution kernels in the first convolutional layer with parameter-learnable Gabor kernels. The dimensions of the feature matrix output by the three convolution layers are 128 × 62 × 62, 128 × 31 × 31 and 128 × 16 × 16, respectively, and these features are shown to have good expressiveness and effectiveness for low-level visual areas [32]. The last fully connected layer is used as the linear readout model to realize the linear mapping from the feature space to the voxel space. The GaborNet-VE can achieve excellent prediction performance in early visual areas (V1, V2 and V3). Hence, we take the GaborNet-VE as the first sub-model in the proposed framework.

#### 2.3.2. S2V2V-EM

S2V2V-EM implements bottom-up hierarchical representation from the voxel perspective and finally encodes high-level visual areas. The architecture of S2V2V-EM is shown in Figure 2. We used the GaborNet-VE to obtain the predicted voxel space of each visual area (including V1, V2 and V4, this paper mainly discusses the low-level visual areas V1 and V2) and the predicted voxel space as the voxel space of the source visual area (indicated in green arrows). In addition, to prevent the interference of invalid encoded voxels, we selected the effectively encoded voxels (ρ > 0.27, *p* < 0.001, see Section 2.5 for details) in the source voxel space for the subsequent analysis.

For the hierarchical readout model, we constructed a voxel-to-voxel model (V2VM) to predict the voxel space of the target visual area with the source voxel space. The V2VM is constructed using a simple fully connected neural network. Undoubtedly, the relationship between the different visual areas in the brain is nonlinear, so we use nonlinear mapping in the V2VM. As shown in Figure 2B, the V2VM consists of two fully connected layers and an activation function is added between them. During training, the source voxel space is mapped to a dimension-specific latent space after passing through the first fully connected layer (the dimension is consistent with the number of voxels in the target visual area; for the S1, the numbers of voxels in visual areas V2, V4 and LO are 2083, 1535, and 928, respectively). After passing through the activation function, the voxel space of the target visual area is output through the second fully connected layer.

In the V2VM, we optimized the weight matrix of the fully connected layer to capture the source voxels that have high accuracy in predicting the target voxels [30]. We refer to these voxels as “intimate voxels”, and the opposite as “distant voxels”. Through continuously increasing the focus on these “intimate voxels” during training, the model achieves the overall best predictive performance. Specifically, during the training of the V2VM, the weight matrix of the fully connected layer reflects the correlation between the source voxels and the target voxels to a certain extent. The larger the weight corresponding to a certain source voxel, the closer the relationship between the voxel and the target voxel. Therefore, when the parameters of the V2VM are backpropagated during training, we squared the weight matrix of the fully connected layer. This not only enhances the correlation of “intimate voxels” between the source visual area and the target visual area but also suppresses “distant voxels”, thereby eliminating the interference of irrelevant voxels in the source visual area to a certain extent.

In general, the visual information in the ventral stream mainly follows the rules of being processed layer by layer and is sequentially transmitted upward from V1 through V2 and V4 and reaches higher visual areas (e.g., LO). In addition, studies showed that the processing of visual information is both parallel and hierarchical, and each visual area is richly connected with other visual areas [47]. Therefore, for the process of hierarchical representation in the ventral stream, there is not only information transfer layer-by-layer but also information transfer across visual layers. For example, part of the information in V1 can be conveyed directly to V4 [46]. Therefore, we constructed S2V2V-EM based on different source visual areas according to the bottom-up information flow direction in the ventral stream.

#### 2.3.3. S2F2V-EM

S2F2V-EM is similar to S2V2V-EM, and the GaborNet-VE is used as the first sub-model. The difference is that S2V2V-EM achieves bottom-up hierarchical representation from the feature perspective.

As shown in Figure 2, after completing the encoding training process for each visual area (V1, V2 and V4) with the GaborNet-VE, we froze the weight parameters of the model. Then we extracted the output of the last convolutional layer in the network (indicated in red arrows) as the feature space (the dimension is 32,768) of the source visual area and input it into the hierarchical readout model. The hierarchical readout model in S2F2V-EM is the feature-to-voxel model (F2VM), which was used to map the source feature space to the voxel space of the target visual area. To facilitate the next step to analyze the impact of hierarchical information conveyance perspectives for the model performance, the F2VM is consistent with the V2VM, consisting of two fully connected layers and one activation function.

### 2.4. Control Models

To further evaluate the performance of our proposed model, we compare it with the following two DNN-based control models.

#### 2.4.1. GaborNet-VE

Based on the GaborNet-VE proposed by Cui et al. [32], we replace the linear mapping of features in the readout model to voxels with a nonlinear mapping. In the fine-tuned GaborNet-VE, the structure of the nonlinear readout model is consistent with V2VM and F2VM in S2V2V-EM and S2F2V-EM. It needs to be emphasized that the encoding performance of the fine-tuned GaborNet-VE is better than the GaborNet-VE in high-level visual areas. In the present study, the GaborNet-VE used to compared with our S2V2V-EM or S2F2V-EM is the fine-tuned GaborNet-VE, if there are no special instructions.

In our proposed S2V2V-EM and S2F2V-EM, nonlinear mapping is used in the hierarchical readout model. However, existing work shows that the nonlinear readout model can improve the model’s performance [10]. Thus, to eliminate the interference of the nonlinear mapping used by the nonlinear readout model for the model’s performance, we fine-tuned the original GaborNet-VE.

#### 2.4.2. CNN-EM

The encoding model based on convolutional neural network features (CNN-EM) refers to the method described in [6,15]. Here, the classic convolutional neural network AlexNet is selected as the image feature extractor [18]. We extracted image features from five pooling layers and the first two full-connected layers of the network layers (7 layers in total) via utilizing the pre-trained AlexNet weight and built an encoding model for each visual area. We used ridge regression to realize the linear mapping from the feature space to the voxel space. Finally, the model with the best encoding performance on the training set was selected as the comparison model for this paper.

### 2.5. Prediction Accuracy, Model Effectiveness Evaluation, and Training Strategy

In this paper, all encoding models constructed (GaborNet-VE, CNN-EM, S2V2V-EM, and S2F2V-EM) were estimated using the 1750 pairs training sample and validated on the 120 pairs testing sample. The prediction accuracy of voxel activity was calculated as the Pearson correlation coefficient between the predicted and the measured voxel activity, and its calculation formula is as follows.

(1) $$\rho =cor\left(\gamma ,\hat{\gamma }\right)=\frac{Cov\left(\gamma ,\;\;\;\hat{\gamma }\right)}{\sqrt{Var\left(\gamma \right)\;\cdot \;Var\left(\hat{\gamma }\right)}},$$

where ρ represents the Pearson correlation coefficient between the measured activity γ and the predicted activity $\hat{\gamma }$ of a single voxel in the testing set. We randomly shuffled the sample correspondence between the measured and the predicted voxel activity and recalculated the correlation. We repeat the process 1000 times to construct a null distribution for each voxel. Finally, we defined ρ = 0.27 (*p* < 0.001, randomization test) as the validity threshold for all voxels according to the null distribution. To visually compare the performance of each set of encoding models, we plotted voxel prediction accuracy scatterplots and corresponding number distributions for each high-level visual area (V4 and LO). In addition, we assessed the significance of a model advantage for a certain visual area (percent of voxels with higher prediction accuracy). Specifically, we selected the voxels that can be accurately predicted by the two models and randomly permuted (with 0.5 probability) the prediction accuracy of the model for each voxel, and then we calculated the advantage. We repeated the process 1000 times to get the null distribution of the model advantage. We found that for any two models, an advantage of more than 53% is significant.

The training strategies of our S2V2V-EM and S2F2V-EM consist of two stages, namely a low-level visual encoding stage and a hierarchical information conveyance stage. There are 90 epochs in the training process. In the low-level visual encoding stage (1st–50th epochs) in Figure 2A, the GaborNet-VE is trained in the low-level visual area and extracts the representation space of the model when the performance is the best. In the hierarchical information conveyance stage (50th–90th epochs) in Figure 2B, the representation space is input into the hierarchical readout model to obtain the encoding result. In addition, we used the Adam optimization algorithm (learning rate is 0.001) to optimize our model, and the batch size was set to 64. Here, the proposed S2V2V-EM and S2F2V-EM are implemented in Pytorch 1.9.1. The training time was about 50 min on a computer with a Nvidia GeForce RTX 3090 graphics card.

### 2.6. Analysis of the Role of the Hierarchy of Representations in Encoding Different Visual Areas

To analyze the role of the hierarchy of representations in the ventral stream for encoding different visual areas (V2, V4, and LO), we calculated the proportion of the best-encoded voxels in different encoding models. Specifically, for the same target visual area, we first selected the voxels in the visual area that could be effectively encoded by at least one of the GaborNet-VE and a different S2V2V-EM (or S2F2V-EM). Then we selected the model with the highest encoding accuracy for each voxel and considered the voxel as the best-encoded voxel for that the model. We drew a pie chart based on the proportion. Take the target visual area V4 as an example; we calculated the best-encoded voxel proportion for the GaborNet-VE and S2V2V-EM (or S2F2V-EM) with V1 and V2 as source visual areas. For the target visual area LO, we additionally constructed S2V2V-EM with V4 as the source visual area for a more comprehensive analysis.

## 3. Results

### 3.1. Comparison of Prediction Accuracy between S2V2V-EM and Control Models

**Figure 3:**
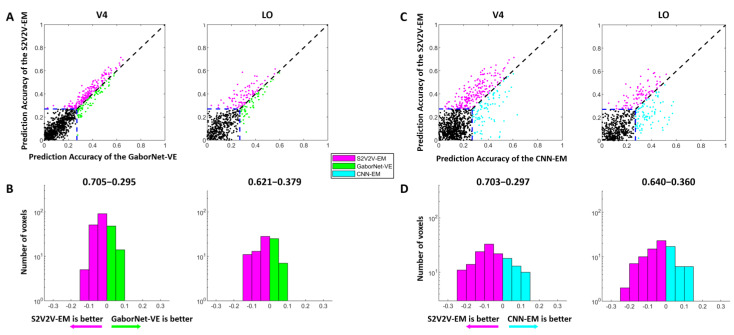
The encoding results of S2V2V-EM, GaborNet-VE, and CNN-EM. (**A**) Comparison of prediction accuracy between S2V2V-EM and GaborNet-VE. Each dot in each sub-figure represents a voxel. The abscissa and ordinate of the dots represent the voxel prediction accuracy of S2V2V-EM and GaborNet-VE, respectively. The blue dashed line represents the validity threshold for prediction accuracy (ρ = 0.27, *p* < 0.001, randomization test). The purple dots represent voxels that can be better encoded by S2V2V-EM, while green dots are the opposite. The black dots represent voxels that could not be efficiently encoded by two models. (**B**) Distribution of prediction accuracy difference between S2V2V-EM and GaborNet-VE. The abscissa and ordinate represent the difference interval of the prediction accuracy and the corresponding number of voxels, respectively. The percentage on each side above the figure represents the proportion of voxels whose prediction accuracy is higher under that model. It should be noted that the voxels used here for comparison can be effectively encoded by two models. (**C**,**D**) are the comparison of S2V2V-EM and CNN-EM. Here, except that the GaborNet-VE compared with S2V2V-EM is replaced with CNN-EM (indicated in cyan), and the detailed description of figures is consistent with (**A**,**B**), respectively.

We constructed different S2V2V-EM based on different source visual areas. Since there are noise voxels in each visual area, we selected the 300 voxels with the highest prediction accuracy (Top-300 voxels) from the predicted voxels for each model, and calculated the average accuracy (Top-300 AA; AA represents the average accuracy). The final results are shown in Table 1. It should be noted that when the source and the target visual area are consistent, the values in the table correspond to the results of the GaborNet-VE. From Table 1, for the target visual area V2, we find that the S2V2V-EM based on the V1 is inferior to GaborNet-VE in performance. However, for target visual areas V4 and LO, all S2V2V-EM are better than GaborNet-VE. For a detailed comparison, we refer to Table 1 to select the S2V2V-EM with the highest average prediction accuracy of the Top-300 voxels in V4 and LO and compare it with the GaborNet-VE and the CNN-EM (see Figure 3).

The comparison of the encoding performance of the S2V2V-EM and the GaborNet-VE is shown in Figure 3A,B. In the scatter plot (Figure 3A), we find that the purple dots are significantly more numerous than the green dots, which shows the S2V2V-EM has obvious advantages over the GaborNet-VE for both V4 and LO. In addition, the number distribution (Figure 3B) indicates that the S2V2V-EM has about 62–70% advantage proportion in the effectively encoded voxels by the two models.

The comparison of encoding performance between the S2V2V-EM and the CNN-EM is shown in Figure 3C,D. For the visual area V4, we find that the S2V2V-EM shows obvious advantages compared to the CNN-EM from the scatter plot and number distribution map. For the visual area LO, the scatter plot shows that the performance of the two models is slightly different. However, the S2V2V-EM has a more significant advantage in the number distribution, which more accurately predicts voxel activity in the voxels that are effectively encoded by both.

Combining the above results, compared with the GaborNet-VE and the CNN-EM, we find that S2V2V-EM achieves the best encoding performance in V4 and LO.

### 3.2. Comparison of Prediction Accuracy between S2F2V-EM and Control Models

**Figure 4:**
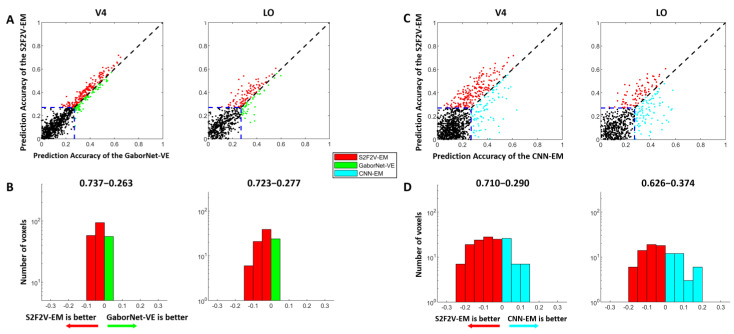
The encoding results of S2F2V-EM, GaborNet-VE, and CNN-EM. The details in the figure are consistent with Figure 3, except that the models compared with GaborNet-VE and CNN-EM are replaced by S2F2V-EM (indicated in red). (**A**,**B**) are the comparison of S2F2V-EM and GaborNet-VE, and (**C**,**D**) are the comparison of S2F2V-EM and CNN-EM.

Similar to S2V2V-EM, we constructed S2F2V-EM based on different source visual areas and calculated the average prediction accuracy of Top-300 voxels for each model. The final results are shown in Table 2. As can be seen from Table 2, for the target visual area V2, we find that the S2F2V-EM is inferior to the GaborNet-VE in performance. However, for target visual areas V4 and LO, all S2F2V-EM are better than GaborNet-VE. We also refer to Table 2 to select the S2F2V-EM with the highest average prediction accuracy of Top-300 voxels in V4 and LO, and compare it with the GaborNet-VE and the CNN-EM (Figure 4).

The comparison of encoding performance between the S2F2V-EM and the GaborNet-VE is shown in Figure 4A,B. In the scatter plot (Figure 4A), we find that the S2F2V-EM is significant for both V4 and LO compared to the GaborNet-VE. In addition, the number distribution (Figure 4B) shows that the proportion of S2F2V-EM advantage voxels exceeded 72%. The comparison of encoding performance of the S2F2V-EM and the CNN-EM is shown in Figure 4C,D. For the visual area V4, we find that the S2F2V-EM shows significant advantages over the CNN-EM. The performance of the two models in the visual area LO is slightly different. Collectively, compared with the GaborNet-VE and the CNN-EM, we find that S2F2V-EM achieves the best encoding performance in V4 and LO.

### 3.3. The Proportion of Best-Encoded Voxels for Different Encoding Models

**Figure 5:**
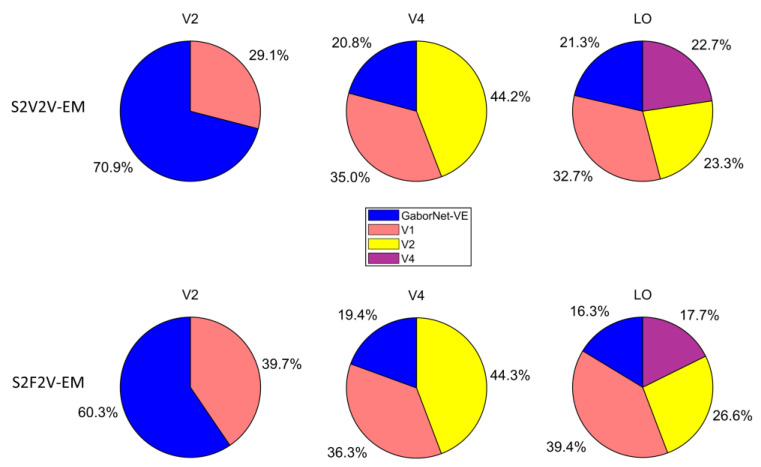
The proportion of best-encoded voxels for different encoding models. The first row represents the comparison of different S2V2V-EM and GaborNet-VE. The second row represents the comparison of different S2F2V-EM and GaborNet-VE. In each sub-figure, GaborNet-VE is illustrated in blue, and S2V2V-EM (or S2F2V-EM) is based on source visual areas; V1, V2, or V4 are in orange, yellow, and purple, respectively. Here, the voxels used for comparison can be accurately predicted by at least one of these models.

The proportion of best-encoded voxels for different encoding models is shown in Figure 5. For the visual area V2, most of the voxels are best encoded by the GaborNet-VE. For the visual area V4, most of the voxels are best encoded by S2V2V-EM based on V1 and V2. For the visual area LO, most of the voxels are best encoded by S2V2V-EM based on V1, V2, and V4. In addition, S2F2V-EM and S2V2V-EM are similar in their results. These results show that the encoding models based on the hierarchical ventral stream-optimization neural network have obvious advantages in high-level visual areas, which can more accurately predict the voxel activity compared with the GaborNet-VE. However, the performance in the V2 is not as good as that of the GaborNet-VE, which suggests that the hierarchy of representations in the ventral stream cannot improve the model’s performance in low-level visual areas.

## 4. Discussion

### 4.1. Advantages of the Encoding Model Framework

The hierarchy of representations in the ventral stream reveals that higher visual areas receive information from lower visual areas. Based on this mechanism, we propose a novel encoding model framework with hierarchical ventral stream-optimized neural networks to improve the performance of the existing model in high-level visual areas. Under the framework, we constructed two categories of hierarchical encoding models, namely S2V2V-EM and S2F2V-EM. Experimental results show (see Figure 3 and Figure 4) that S2V2V-EM and S2F2V-EM accurately predict most voxel activity in V4 and LO compared to the GaborNet-VE [32]. Therefore, the framework can exploit the advantages of the existing encoding model for low-level visual areas to improve the model’s performance in high-level visual areas. Compared with the DNN-based encoding model (i.e., CNN-EM) [6,15], S2V2V-EM and S2F2V-EM also show significant advantages in V4 and LO, which further demonstrates the framework’s value in encoding high-level visual areas. Moreover, the proportions of best-encoded voxels for different models (see Figure 5) indicate that S2V2V-EM (or S2F2V-EM) has a significant advantage in V4 and LO, and most of the voxels (about 80%) of V4 and LO are best-encoded by different S2V2V-EM (or S2F2V-EM). Importantly, the best-encoded voxels are distributed in the S2V2V-EM (or S2F2V-EM) based on different source visual areas, which proves that the advantages of the framework primarily stem from the hierarchy of representations in the ventral stream. Collectively, the encoding models under the framework utilize the hierarchy of representations in the ventral stream to effectively improve the performance of the existing model in high-level visual areas. In addition, the direction of hierarchical information conveyance in the framework is in line with the known topological connections of the ventral visual areas [46]; thus, the framework has a certain biological rationality.

### 4.2. The Effects of Hierarchical Information Conveyance Perspectives on Model Encoding Performance

The key principle for our proposed framework is to extract the representation space of the encoding model trained at the low-level visual area and further realize the encoding of high-level visual areas. Therefore, for the framework, the choice of the representation space is important. Undoubtedly, the feature space of images obtained by the model is a choice, and it is the choice for almost all encoding models. In addition, inspired by Mell et al. [43], we assume that the voxel space predicted by the model is the measured voxel space and use the former as another option for the representation space.

Finally, we realize the bottom-up process of hierarchical information conveyance from the voxel and the feature perspectives. Our experimental results show that the encoding models constructed based on the above two perspectives can effectively improve the model’s performance in high-level visual areas. However, in comparison with the GaborNet-VE, we find that S2F2V-EM (corresponding to the feature perspective) has a more significant advantage than S2V2V-EM (corresponding to the voxel perspective) in high-level visual areas. On the one hand, we speculate that the reason for this discrepancy is the information decay caused by mapping the feature space to the voxel space in the encoding model (GaborNet-VE) because, in this study, the dimension of the feature space extracted from the model is much larger than that of the predicted voxel space. Therefore, the feature space may contain more representation information to the source low-level visual area. Another reason may be that the predicted voxel space is different from the measured voxel space (i.e., at present, for most voxels in a specific visual area, the Pearson correlation coefficient between the measured and predicted voxel activity will not be 1 or ~1), resulting in the accuracy that uses the predicted voxel space to predict the voxel space of high-level visual areas being not as good as using the measured voxel space [43]. In summary, we speculate that the above two reasons lead to the difference in the final performance of the encoding models based on the two perspectives of hierarchical information conveyance.

### 4.3. The Encoding Contribution of Different Low-Level Visual Areas to the High-Level Visual Area

In the ventral stream, the same source visual area is projected to different target visual areas, and the same target visual area receives information from different source visual areas. However, the weights of the connections between visual areas are different, whether as a fraction of the source visual output or as a fraction of the target visual input [48]. Adjacent visual areas are most closely connected, a principle that explains many of the known connections among ventral visual areas [49]. The results of Section 3.3 show the best-encoded voxels are distributed in different S2V2V-EM, which also indicates that the weight of connection is reflected in the encoding contribution of different low-level visual areas to the high-level visual area. Moreover, we found that for the target visual area V4, the S2V2V-EM (or S2F2V-EM) with the best encoding performance originated from V2, not V1, which suggests that V2 may contain more information that helps to encode V4. This is consistent to a certain extent with the conclusion in [43]: the prediction accuracy between voxels in different visual areas decreases as the visual hierarchical distance between the source and target visual area increases. For the topological connection of the ventral stream [46], the hierarchical distance between V2 and V4 is smaller than between V1 and V4. However, for the target visual area LO, we found that the best S2V2V-EM does not come from V4 (closer to LO in the hierarchical distance) but from V1 farther away. The reason for this difference may be that the original encoding model (GaborNet-VE) has insufficient encoding performance for the high-level visual area V4, which causes the S2V2V-EM (or S2F2V-EM) to select the low-level visual area V1 with better encoding performance.

### 4.4. Limitations and Future Work

Although our model framework has effects in improving the performance of existing models in high-level visual areas, this framework still has several disadvantages. The results demonstrate that the framework has no effect in low-level visual areas (see Figure 5) and relies on the existing visual encoding model as a bridge between higher and lower visual areas. On the other hand, our framework only considers the interaction between single visual areas (e.g., V1→V4 or V2→V4) but multiple visual areas are not fully considered (e.g., V1+V2→V4). Future work will use this framework to investigate the contribution of voxels in different source visual areas to target voxels and select the best source voxel to achieve the comparatively best encoding effect for each target voxel.

## 5. Conclusions

We proposed a novel model framework which optimizes existing encoding models by exploiting the hierarchy of representations in the ventral stream. Based on the framework, we constructed two categories of hierarchical encoding models from the voxel and the feature perspectives for high-level visual areas. Experimental results show that the encoding models under the framework can effectively improve the performance of the existing encoding model in high-level visual areas. Therefore, we find that the hierarchy of representations in the ventral stream has positive significance for optimizing the existing encoding model.

## Figures and Tables

**Table 1:** The average prediction accuracy of Top-300 voxels for the GaborNet-VE and different S2V2V-EM.

Source	Target	Top-300 AA
V1	V2	0.6284
V2		**0.656** **1**
V1	V4	0.3731
V2		**0.3761**
V4		0.3540
V1	LO	**0.2647**
V2		0.2594
V4		0.2626
LO		0.2234

The best results on each target visual area are in bold.

**Table 2:** The average prediction accuracy of Top-300 voxels for the GaborNet-VE and different S2F2V-EM.

Source	Target	Top-300 AA
V1	V2	0.6411
V2		**0.656** **1**
V1	V4	0.3717
V2		**0.3771**
V4		0.3540
V1	LO	**0.2673**
V2		0.2645
V4		0.2577
LO		0.2234

The best results on each target visual area are in bold.

## Data Availability

The detailed information about the fMRI data is provided in previous studies, and the public dataset can be downloaded from https://crcns.org/data-sets/vc/vim-1 (accessed on 27 June 2022).
